# Plasma p-tau217, p-tau181, and Aβ42 Predicts Amyloid PET Positivity in Cognitively Unimpaired Adults

**DOI:** 10.21203/rs.3.rs-8587114/v1

**Published:** 2026-01-20

**Authors:** Rui Bao, Wanying Shi, Hongbo Bao, Tonghua Zhang, Xueying Li, Wencai Ding

**Affiliations:** The Second Affiliated Hospital of Wannan Medical College; Department of Neurology The First Affiliated Hospital, and College of Clinical Medicine of Henan University of Science and Technology; Department of Neurosurgery, Beijing Tiantan Hospital, Capital Medical University; The Second Affiliated Hospital of Wannan Medical College; Department of Neurology, The First Affiliated Hospital of Dalian Medical University; The Second Affiliated Hospital of Wannan Medical College

**Keywords:** p-tau217, Alzheimer's disease, Plasma biomarkers, Cognition unimpaired

## Abstract

**Background:**

Early detection of Alzheimer's disease (AD) pathology in cognitively unimpaired individuals is critical for preclinical intervention. Plasma biomarkers, especially phosphorylated tau217 (p-tau217), are promising predictors of amyloid-β (Aβ) accumulation.

**Methods:**

In this cohort study, we analyzed data from cognitively unimpaired older adults in the A4 and LEARN studies (n=1,407), comprising 452 participants with Aβ positron emission tomography (PET)-negative status and 955 participants with Aβ PET-positive status. We evaluated the accuracy of plasma biomarkers (p-tau217, p-tau181, Aβ42/40 ratio, and others) in predicting Aβ PET positivity using receiver operating characteristic analysis, comparing models with biomarkers alone versus those combined with covariates (age, sex, apolipoprotein E [APOE] ε4 genotype).

**Results:**

Plasma p-tau217 showed the strongest individual association with Aβ PET status (area under the curve [AUC] 0.85). A combined model integrating p-tau217, p-tau181, Aβ42, age, sex, and APOE ε4 achieved the highest diagnostic accuracy (AUC 0.87), significantly outperforming individual biomarkers.

**Conclusions:**

Plasma p-tau217, particularly when combined with other biomarkers and clinical covariates, provides a robust method for predicting Aβ PET positivity in cognitively unimpaired older adults. This biomarker profile could enhance preclinical trial screening by identifying individuals likely to harbor Aβ pathology, potentially reducing the need for confirmatory PET scans.

## Background

As disease-modifying therapies (DMTs) move into clinical use, the focus of therapeutic intervention is increasingly shifting to the preclinical stage of AD, where Aβ accumulation occurs in the absence of cognitive decline[1]. Early detection and intervention during this phase hold the potential to delay or prevent the onset of AD-related cognitive impairment[2]. However, current gold-standard diagnostic methods, including cerebrospinal fluid (CSF) analysis and PET, remain invasive, costly, and logistically challenging for widespread use in prevention trials. Blood-based biomarkers (BBMs) offer a promising, less invasive alternative for confirming Aβ pathology, providing a scalable approach to identify individuals likely to harbor cerebral Aβ pathology for preclinical trial screening[1, 3-6].

Among BBMs, plasma phosphorylated tau species, particularly p-tau217, and the Aβ42/Aβ40 ratio have shown strong correlations with Aβ burden and have been proposed as potential tools for early detection of AD[7-12]. Previous studies have demonstrated that p-tau217 correlates strongly with PET and CSF biomarkers, suggesting its potential as an early indicator of AD-related pathophysiology[13-15]. Moreover, p-tau217 has been shown to predict cognitive decline in preclinical populations, making it a promising biomarker for participant selection in clinical trials[14, 16, 17]. However, while these biomarkers hold promise, there is still a need for systematic evaluation of their diagnostic performance, particularly within preclinical cohorts enriched for amyloid positivity and in cognitively unimpaired populations, where conventional performance thresholds may differ.

To address these gaps, we conducted a cohort study of cognitively unimpaired older adults from the A4/LEARN study to compare the diagnostic accuracy of six plasma biomarkers, including p-tau217 and the Aβ42/Aβ40 ratio, for predicting Aβ PET positivity. Specifically, this study aimed to (1) evaluate the diagnostic performance of individual biomarkers and their ratios; (2) assess associations with continuous Aβ PET burden; and (3) investigate the utility of combining biomarkers with demographic and genetic covariates to improve classification accuracy. These analyses provide critical insights into the potential role of plasma biomarkers in trial screening and enrich for Aβ PET positivity, facilitating the development of more efficient preclinical trial recruitment strategies.

## Methods

### Study Participants

This study used data from the A4 Study and the LEARN Study[18], which were designed based on the amyloid hypothesis of AD, positing Aβ accumulation as an early and central pathogenic process. Participants were community-dwelling older adults aged 65-85 years who were clinically unimpaired at baseline, with no diagnosis of mild cognitive impairment or dementia. Individuals with unstable medical or psychiatric conditions were excluded, while those with stable comorbidities such as hypertension, diabetes, or hyperlipidemia were eligible. All participants provided written informed consent, and each was required to have a study partner familiar with their daily functioning who also consented to participate.

### Amyloid Status Definition

Baseline cerebral amyloid burden was assessed using ^18^F-florbetapir PET imaging in participants from the A4 and LEARN studies. Image acquisition and processing followed established study-specific procedures. Aβ burden was quantified using a cortical composite standardized uptake value ratio (SUVR) generated with PMOD software, with normalization to cerebellar gray matter[19]. Amyloid status was determined using PET-derived composite SUVR thresholds applied during the A4 screening process. Participants with SUVR values of 1.15 or greater were classified as amyloid positive without additional qualitative review[20]. For scans with intermediate SUVR values (1.10-1.15), amyloid status was adjudicated based on independent visual assessment by certified nuclear medicine physicians.

### Plasma Biomarker Measurements

Plasma biomarkers were quantified through the A4 multilaboratory biomarker consortium. Concentrations of Aβ40 and Aβ42, including free, total, and bound fractions, were measured by Araclon Biotech. p-tau217 was measured by Eli Lilly using an electrochemiluminescence immunoassay automated on a Tecan Fluent workstation, with signal detection performed on an MSD Sector S 600MM imager[21]. In addition, plasma samples were analyzed by Roche Diagnostics using Elecsys prototype electrochemiluminescence immunoassays to quantify Aβ40, Aβ42, and p-tau181[21]. All assays were conducted according to laboratory specific, rigorously validated standard operating procedures. Additional details are included in the eMethods in Supplement 1.

### Statistical Analysis

All statistical analyses were conducted using R software (version 4.4.1). Demographic and clinical characteristics were compared between the Aβ PET-negative and Aβ PET-positive groups using independent-samples t tests or Mann-Whitney U tests for continuous variables and χ^2^ tests for categorical variables. Plasma biomarker concentrations (Aβ42, Aβ40, p-tau181, and p-tau217) were log-transformed to improve normality before parametric analyses.

Differences in plasma biomarker levels between groups were evaluated using generalized linear models, with adjustment for age, sex, and APOE ε4 carriage, as appropriate. Effect sizes were reported as Cohen’s d. Receiver operating characteristic curves were constructed to assess the ability of individual biomarkers and combined models to discriminate Aβ PET status. Area under the curve values with 95% confidence intervals were calculated, and sensitivity, specificity, positive predictive value, negative predictive value, and overall accuracy were estimated at optimal cutoffs determined using the Youden index. Comparisons between ROC curves were performed using the DeLong test[22]. The probability of Aβ PET positivity across the range of biomarker values was estimated using generalized linear models with binary Aβ status as the outcome. For visualization, histogram bars and density curves were scaled to the Aβ probability axis (range, 0-1). Associations between continuous plasma biomarker concentrations and cerebral Aβ burden, expressed as SUVR, were evaluated using linear regression, with the strength of association quantified by the coefficient of determination (R^2^). All statistical tests were 2-sided, with statistical significance defined as P < .05. P values were not adjusted for multiple comparisons; however, when appropriate, results with P values greater than the Bonferroni-adjusted significance threshold (α = 0.001) but less than .05 were interpreted as nominally significant. Multivariable feature selection was performed using the least absolute shrinkage and selection operator (LASSO), incorporating all candidate plasma biomarkers, including biomarker ratios, along with relevant covariates.

## Results

### Demographic and Clinical Characteristics

The study included 1407 cognitively unimpaired participants from the A4/LEARN cohort, comprising 452 Aβ PET-negative and 955 Aβ PET-positive individuals (Table 1). The two groups did not differ significantly with respect to sex distribution or years of education. Participants in the Aβ PET-positive group were older (mean age, 71.9 vs 70.4 years) and had a higher prevalence of APOE ε4 carriage (60.4% vs 22.6%); both differences were statistically significant (*P* < 1 × 10^−4^). Plasma concentrations of all measured biomarkers differed significantly between groups (*P* < 1 × 10^−4^). On cognitive testing, participants in the Aβ PET-positive group had lower Mini-Mental State Examination scores (*P* = 0.03), lower Preclinical Alzheimer Cognitive Composite scores (*P*= 0.0002), and poorer performance on several component tests of the composite. Performance on the Logical Memory delayed recall test did not differ significantly between groups.

### Comparison of Plasma Biomarker Profiles by Aβ PET Status

**Figure 1:**
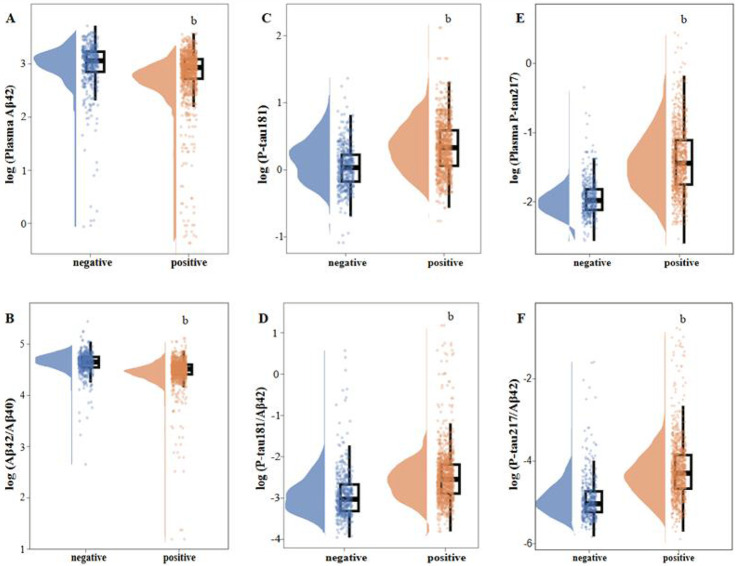
Legend not included with this version.

The distributions of the six log-transformed plasma biomarkers stratified by Aβ PET status are shown in Figure 1, and quantitative comparisons between the Aβ PET-negative and Aβ PET-positive groups are summarized in Table 2. All plasma biomarkers differed significantly between groups (*P*< 0.0001), with effect sizes ranging from moderate to large. Phosphorylated tau biomarkers showed the greatest separation between groups. Plasma p-tau217 demonstrated the largest effect size (Cohen’s d = −1.30), indicating substantially higher levels in Aβ PET-positive individuals. The p-tau217/Aβ42 ratio also showed a large effect size (Cohens’ d = −1.02). Among amyloid-related biomarkers, the Aβ42/40 ratio showed a moderate effect size (Cohen’s d = 0.53), whereas Aβ42 alone exhibited the smallest between-group difference (Cohen’s d = 0.28). Plasma p-tau181 and the p-tau181/Aβ42 ratio showed moderate effect sizes (Cohen’s d = −0.80 and −0.69, respectively). These between-group differences remained materially unchanged after adjustment for age, sex, and APOE ε4 allele status.

### Associations Between Plasma Biomarkers and Aβ PET Burden

**Figure 2:**
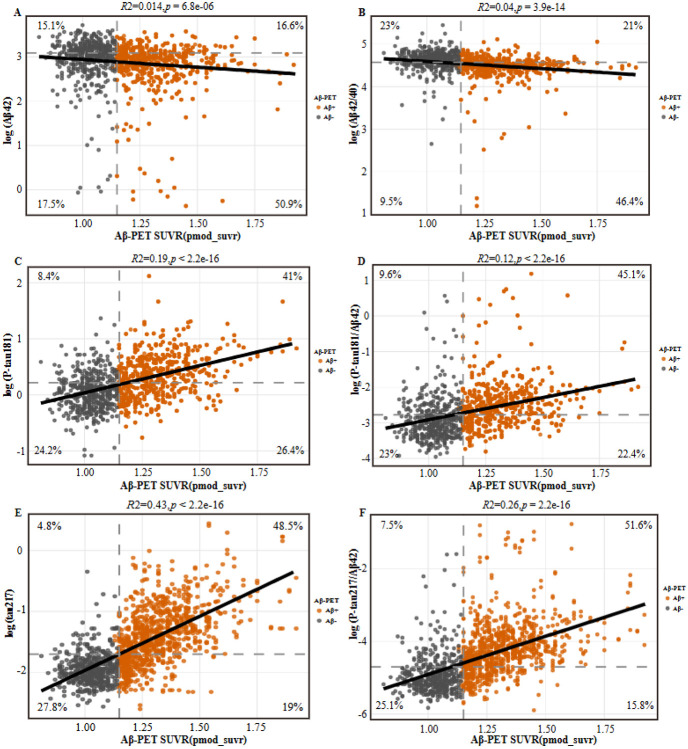
Legend not included with this version.

We next examined associations between log-transformed plasma biomarker concentrations and continuous Aβ PET burden, quantified using SUVR, with the strength of association assessed by linear regression (Figure 2). Among all biomarkers, plasma p-tau217 showed the strongest association with global Aβ PET burden (*R^2^* = 0.43; *P* < 2.2 × 10−^16^). The p-tau217/Aβ42 ratio also demonstrated a strong association (*R^2^* = 0.26; *P* < 2.2 × 10^−16^). In contrast, plasma p-tau181 and the p-tau181/Aβ42 ratio showed more modest associations with Aβ PET SUVR (*R^2^* = 0.19 and 0.12, respectively; both *P* < 2.2 × 10^−16^). Amyloid-related plasma biomarkers showed weaker associations with cerebral Aβ burden. The Aβ42/40 ratio and Aβ42 alone were associated with Aβ PET SUVR with low coefficients of determination (*R^2^*= 0.04 and 0.014, respectively), despite reaching statistical significance (*P*= 3.9 × 10^−14^ and *P*= 6.8 × 10^−6^). Misclassification patterns across biomarkers are illustrated in Figure 2. Plasma p-tau217 and the p-tau217/Aβ42 ratio were associated with lower rates of false-positive and false-negative classifications compared with other biomarkers, whereas p-tau181-based measures showed higher false-negative rates. Amyloid-based plasma measures showed limited discriminative performance, with higher false-positive rates observed for the Aβ42/40 ratio.

### Performance of Plasma Biomarkers for Predicting Aβ Status

**Figure 3:**
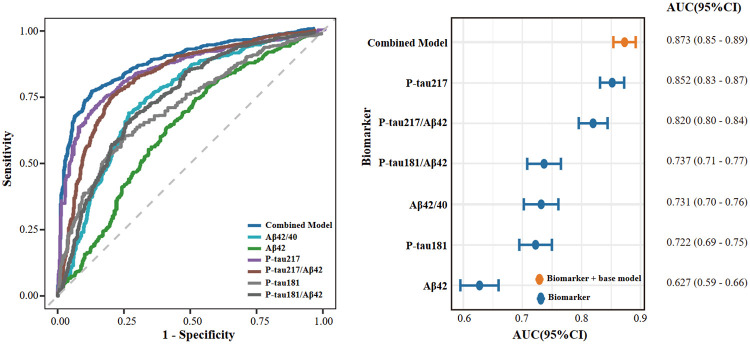
Legend not included with this version.

The diagnostic performance of individual log-transformed plasma biomarkers, biomarker ratios, and a combined model for classifying Aβ PET status was evaluated using receiver operating characteristic analysis. Receiver operating characteristic curves and corresponding area under the curve statistics are shown in Figure 3. The combined model, which included p-tau217, p-tau181, and Aβ42, age, sex, and APOE ε4 allele status, showed the highest discriminative performance (AUC, 0.87; 95% CI, 0.85-0.89). Among individual biomarkers and ratios, plasma p-tau217 demonstrated the strongest performance (AUC, 0.85; 95% CI, 0.83-0.87), followed by the p-tau217/Aβ42 ratio (AUC, 0.82; 95% CI, 0.80-0.84). The Aβ42/40 ratio showed moderate discriminative ability (AUC, 0.73; 95% CI, 0.70-0.76). The performance of p-tau181/Aβ42 and p-tau181 were similar (AUC, 0.74; 95% CI, 0.71-0.77 and AUC, 0.72; 95% CI, 0.69-0.75, respectively). Plasma Aβ42 alone showed the lowest discriminative performance (AUC, 0.63; 95% CI, 0.59-0.66). Overall, combining plasma p-tau217 with amyloid-related biomarkers and clinical covariates improved the classification of Aβ PET status compared with individual biomarkers alone.

### Accuracy and classification Metrics of Plasma Biomarkers Using Single Cutoffs

Using optimal cutoffs derived from the Youden index, we evaluated the diagnostic performance of individual log-transformed plasma biomarkers and their ratios for predicting Aβ PET positivity in cognitively unimpaired older adults (Table S1 and Figure S1). The p-tau217/Aβ42 ratio showed the highest accuracy (76.69%) and a balanced sensitivity and specificity profile (76.34% and 77.43%, respectively), followed by plasma p-tau217 alone (accuracy, 76.26%; specificity, 85.84%). Other biomarkers, including the Aβ42/40 ratio and the p-tau181/Aβ42 ratio, showed moderate accuracy (69.58% and 68.16%, respectively). Plasma Aβ42 alone showed the lowest accuracy (66.10%) and specificity (46.46%). A combined model incorporating p-tau217, p-tau181, and Aβ42, along with age, sex, and APOE ε4 allele status, showed improved overall performance (accuracy, 80.03%; AUC, 0.87), with higher specificity (86.28%) and positive predictive value (92.23%) than any individual biomarker or ratio. Overall, p-tau217-based measures showed consistent performance for classifying amyloid status in this preclinical cohort, whereas integration of multiple plasma biomarkers with demographic and genetic factors further improved diagnostic performance.

## Discussion

The main results of this cohort study suggest that p-tau217-based plasma measures outperform other plasma biomarkers for identifying ^18^F-florbetapir Aβ PET positivity in cognitively unimpaired older adults. We compared associations with continuous Aβ PET SUVR and classification performance across 6 plasma biomarkers and biomarker ratios. First, p-tau217 showed the strongest association with global Aβ PET burden, and p-tau217 and the p-tau217/Aβ42 ratio showed the largest between-group differences. Second, p-tau217-based measures provided the highest discrimination of Aβ PET positivity; however, negative predictive values were modest in this screening-enriched preclinical sample, limiting rule-out utility when used in isolation. Third, a combined model integrating p-tau217 with p-tau181 and Aβ42, together with age, sex, and APOE ε4 status, provided the best overall classification performance, supporting a blood-based prescreening strategy to enrich for Aβ PET positivity and reduce the number of confirmatory PET scans needed for screening.

A key implication of these findings is that p-tau217-based measures may capture early downstream responses to cerebral amyloid accumulation more reliably than plasma amyloid peptide measures in cognitively unimpaired individuals. Prior work has consistently shown that p-tau217 performs strongly across platforms and clinical stages, supporting its value as an early indicator of Alzheimer disease-related pathophysiology[7, 23]. In a screening-enriched cohort such as A4/LEARN, plasma Aβ measures may show a narrower dynamic range and may be more sensitive to preanalytical and assay-related variation, which could attenuate their discrimination when amyloid burden is modest and participants remain clinically normal[24]. By contrast, p-tau217 may reflect amyloid-associated tau phosphorylation that becomes detectable even when cognitive performance is largely preserved, thereby yielding a clearer blood-based signal aligned with Aβ PET[8, 25]. This interpretation helps reconcile why p-tau217 outperformed Aβ42 and Aβ42/40 in our cohort while remaining consistent with studies that reported stronger amyloid-peptide performance in samples spanning broader disease severity[24]. Together, these data support using p-tau217-centered plasma profiles as a practical first-line enrichment tool in preclinical trial screening while acknowledging that confirmatory amyloid assessment remains necessary when the goal is to definitively establish Aβ status.

In practical terms, our findings support a stepwise “blood-first” prescreening approach for prevention-oriented pipelines, in which p-tau217-centered measures (alone or combined with complementary markers and readily available covariates) are used to enrich for Aβ PET positivity before confirmatory testing[26]. This framing aligns with recent recommendations that blood-based biomarkers may have their greatest near-term impact when deployed to reduce the number of expensive or less accessible confirmatory procedures, rather than as stand-alone diagnostic substitutes in asymptomatic populations[27, 28]. Importantly, the modest negative predictive values observed in our cohort should be interpreted in the context of the A4/LEARN screening design, where the prevalence of Aβ positivity is high and inevitably constrains rule-out performance even for otherwise strong assays; accordingly, single-marker strategies may be better suited for enrichment (rule-in) than for excluding amyloid pathology in such settings[9, 29, 30]. One practical way to address this limitation is to prespecify operating points tailored to the intended use case, including dual-threshold algorithms that create an intermediate “gray zone” requiring confirmatory PET (or CSF), while allowing high-confidence rule-in and rule-out decisions at the extremes. Recent work evaluating combined p-tau217 and amyloid measures on fully automated platforms demonstrates that dual cutoffs can yield high overall accuracy while leaving a manageable minority of participants in the intermediate zone, providing a concrete template for implementation and triage workflow[7, 31, 32]. Finally, translation will depend on analytical robustness and portability: accumulating evidence from head-to-head comparisons, cut-point optimization studies, and implementation frameworks indicates that assay design and platform-specific calibration can meaningfully influence performance estimates and threshold transferability, reinforcing the need for external validation of any proposed prescreening algorithm across laboratories and more diverse populations before clinical deployment[14, 25, 33, 34].

### Limitations

This study has several limitations. First, the cross-sectional design limits causal inference and precludes evaluation of how plasma biomarkers predict longitudinal amyloid accumulation or subsequent cognitive change; prospective follow-up will be needed to establish prognostic utility. Second, the A4/LEARN cohort comprises highly screened, generally healthy volunteers and is predominantly White, which may limit generalizability to more clinically and demographically diverse populations and to settings with lower amyloid prevalence. Third, we defined classification performance using a single “optimal” cutoff derived from the Youden index. Although this approach provides a consistent benchmark for head-to-head comparisons, it may not reflect the operating points most relevant for trial prescreening, where investigators often prioritize either sensitivity (to minimize missed Aβ positivity) or specificity (to reduce confirmatory scans). Relatedly, negative predictive values were modest in this enrichment cohort, and misclassification may be more common among individuals near the PET threshold. Fourth, plasma biomarkers were measured across different laboratories and assay platforms, and although each followed validated procedures, interplatform differences and preanalytical variability may affect absolute values and threshold portability. Finally, the combined model was developed and evaluated within the same cohort; model performance may therefore be optimistic, and external validation is required to confirm generalizability and the stability of coefficients and cut points before implementation in other trial pipelines.

## Conclusion

In this cohort study of cognitively unimpaired older adults screened in A4/LEARN, p-tau217-based plasma measures showed the most consistent performance for identifying ^18^F-florbetapir Aβ PET positivity, and a multivariable model that integrated p-tau217 with p-tau181, Aβ42, and key covariates further improved classification. These findings support blood-based prescreening as an enrichment tool for prevention-trial recruitment, while underscoring that confirmatory assessment remains necessary when definitive amyloid status is required. Future work should validate these prescreening algorithms in independent and more diverse cohorts, evaluate prespecified operating points (including potential dual-threshold strategies), and determine longitudinal and real-world screening performance in prevention-oriented trial workflows.

## Supplementary Material

This is a list of supplementary files associated with this preprint. Click to download.

- Supplement1.docx
- Supplement2.docx

## Figures and Tables

**Table 1.** Sample clinical and demographic characteristics in CU individuals.

	All clinically unimpaired A− (N=452)	All clinically unimpaired A+ (N=955)	P-value
Sex (F), n (%)	173 (38.3%)	382 (40.0%)	0.575
Age, years	70.4 (4.28)	71.9 (4.86)	<0.0001
Education, years	16.7 (2.65)	16.6 (2.54)	0.482
APOE ε4 carriage, n (%)	102 (22.6%)	577 (60.4%)	<0.0001
**Cognition**			
PACC	−0.633(2.21)	−1.12 (2.45)	0.000186
MMSE	28.9 (1.11)	28.8(1.27)	0.0271
MMSE (z-score)	0.129(0.867)	0.0146(0.992)	0.0271
FCSRT96 (z-score)	−0.106(0.919)	−0.231(0.995)	0.0203
DSC (z-score)	−0.446(0.849)	−0.597(0.885)	0.0022
LMDR (z-score)	−0.210(0.922)	−0.309(0.922)	0.059
**Biomarker**			
Aβ42	21.0 (6.89)	18.3 (6.15)	<0.0001
Aβ42/40	104 (21.0)	90.2 (18.5)	<0.0001
P-tau181	1.10 (0.401)	1.52 (0.702)	<0.0001
P-tau181/Aβ42	0.0736 (0.133)	0.127 (0.255)	<0.0001
P-tau21	0.149 (0.0532)	0.279 (0.165)	<0.0001
P-tau217/Aβ42	0.00977 (0.0165)	0.0229 (0.0425)	<0.0001

**Note:** The cutoff for Aβ PET positivity was defined as an SUVR of 1.15 or greater. Continuous variables are presented as mean (SD), and categorical variables are presented as number (percentage).

**Abbreviations:** Aβ PET, amyloid-β positron emission tomography; Aβ, amyloid-β; APOE, apolipoprotein E; CU, cognitively unimpaired; DSC, Digit Symbol Coding Test; FCSRT, Free and Cued Selective Reminding Test; LMDR, Logical Memory Delayed Recall; MMSE, Mini-Mental State Examination; PACC, Preclinical Alzheimer Cognitive Composite; PET, positron emission tomography; p-tau, phosphorylated tau; SD, standard deviation.

**Table 2:** Comparison of plasma biomarker levels between Aβ-PET groups.

Biomarker	Mean (SD) PET-Aβ−	Mean (SD) PET-Aβ+	Cohen’s D	*P* value^a^	*P* value^b^
**Aβ42**	21.0 (6.89)	18.3 (6.15)	0.282	8.91E-7	2.39E-04
**Aβ42/40**	104 (21.0)	90.2 (18.5)	0.529	7.54E-20	1.76E-11
**P-tau181**	1.10 (0.401)	1.52 (0.702)	−0.803	3.76E-42	3.60E-27
**P-tau181/Aβ42**	0.074 (0.133)	0.127 (0.255)	−0.693	2.43E-32	1.59E-19
**P-tau217**	0.149 (0.053)	0.279 (0.165)	−1.30	2.45E-97	3.37E-67
**P-tau217/Aβ42**	0.010 (0.017)	0.023 (0.043)	−1.02	7.60E-65	2.97E-42

**Note:** Data are presented as mean (SD) for log-transformed biomarker values. The cutoff for Aβ PET positivity was defined as an SUVR of 1.15 or greater.

**Abbreviations:** Aβ, amyloid-β; PET, positron emission tomography; SD, standard deviation; SUVR, standardized uptake value ratio. *P*value^a^: *P*value derived from comparison of means using log-transformed biomarker data without adjustment for age, sex, APOE ε4 allele status, or PET tracer. *P* value^b^: *P*value derived from comparison of means using log-transformed biomarker data with adjustment for age, sex, APOE ε4 allele status.

## Data Availability

See Supplement 2.
